# Xanthan biopolymer-based soil treatment effect on kaolinite clay fabric and structure using XRD analysis

**DOI:** 10.1038/s41598-023-38844-w

**Published:** 2023-07-19

**Authors:** Yeong-Man Kwon, Ilhan Chang, Gye-Chun Cho

**Affiliations:** 1 Department of Civil and Environmental Engineering, Northwestern University, Evanston, IL 60208 USA; 2 Department of Civil Systems Engineering, Ajou University, Suwon, 16499 Republic of Korea; 3 Department of Civil and Environmental Engineering, Korea Advanced Institute of Science and Technology (KAIST), Daejeon, 34141 Republic of Korea

**Keywords:** Biogeochemistry, Engineering

## Abstract

In this study, we evaluated the impact of xanthan gum biopolymer (XG) on kaolinite fabrics using X-ray diffraction (XRD) and the ensuing changes in the compaction behavior and shear resistance of kaolinite soils. The XRD peak analysis revealed that XG changed kaolinite fabrics into face-to-face associations. Moreover, environmental scanning electron microscopy showed the formation of XG-bridges between kaolinite particles, resulting in the change in fabrics and subsequently improving the resistance of kaolinite to external forces. Consequently, as XG content increased, the maximum dry density decreased, and the undrained shear strength increased. The viscous XG hydrogels produced a higher optimal moisture content and increased resistance to shear force. This study showed that XG affects the mechanical properties of kaolinite through changing kaolinite fabrics (up to 0.5% of the XG-to-kaolinite mass ratio) and absorbing pore-fluids (excess XG over 0.5% of the XG-to-kaolinite mass ratio).

## Introduction

The global carbon dioxide (CO_2_) emissions and the increasing global warming have resulted in catastrophic climate change, including rising sea levels, heat waves, and rainfall imbalances^1–3^. To mitigate the concerns surrounding climate change, geotechnical engineers have conducted various studies to develop eco-friendly materials to replace the use of ordinary Portland cement (OPC) in soil improvement materials^4^. The OPC accounts for 5%–7% of anthropogenic CO_2_ emissions with one ton of CO_2_ emission for every ton of production^5,6^.

The biopolymer-based soil treatment (BPST), which uses exo-cultured biopolymers resulting from the metabolism of living organisms for soil improvement, has been extensively studied for integration as an environmentally friendly soil improvement technique^7–9^. The biopolymers affect soil grains by increasing the pore-fluid viscosity and particle interaction^10^. Based on the interaction of biopolymers and soils, biopolymers have shown their ability to enhance soil consistency^11^, coagulation^12^, soil strength^13–18^, surface erosion^19–22^, and hydraulic conductivity control^23–26^.

In the context of clays, the presence of biopolymers can lead to direct ionic bonding with the surface of clay particles^27^, resulting in modifications of the clay fabric, and geometric arrangement of the soil particles^10,11^. For instance, Mahamaya et al.^28^ investigated the effect of xanthan gum (XG), guar gum, and cellulose biopolymers on fly ash and mine tailings. The study demonstrated improvements in index properties, compressive strength, and water erosion resistance, attributed to morphological changes due to the interaction between the soil and the long-chained biopolymer strings. Similarly, Hamza et al.^29^ found that XG-induced clay aggregation enhanced geotechnical parameters of soils such as strength, consolidation, hydraulic conductivity, and freeze–thaw durability. Kang et al.^30^ compared the effects of various biopolymers on the settling characteristics of kaolinite and fly ashes, revealing that cationic biopolymers induced fabric changes in kaolinite through bridging and charge neutralization, resulting in an increased settling velocity. Microscopic techniques, including scanning electron microscopy (SEM) and energy-dispersive X-ray spectroscopy (EDS), have been widely utilized in previous research to investigate the interactions between biopolymers and clays. However, electron microscopy techniques have practical limitations, including challenges in sample preparation and limited surface observation^31^, which hinder a comprehensive understanding of the biopolymer’s effect on the fabric of bulk clay materials.

To bridge this knowledge gap, this study aims to examine and analyze the fabric of XG biopolymer-treated kaolinite by employing X-ray diffraction (XRD) technique, as well as variations in its mechanical properties. XRD technique is extensively utilized for analyzing the structure of bulk clays due to its capability to probe depths of 30–50 μm^32,33^. Among a variety of organic polymers^30^, XG was selected in this study owing to its wide usage in various applications^34–36^, promising results in enhancing geotechnical properties^13,37,38^, and unique rheological properties such as the formation of viscous hydrogels and pseudoplastic behavior^39,40^. These characteristics make XG a suitable candidate for investigating its effects on the fabric and mechanical properties of kaolinite clay. Several experimental methods were used to achieve the study’s goal. The XRD patterns of XG-treated kaolinites were obtained to determine the fabric of clays by comparing the X-ray peaks and their amplitudes. By integrating SEM and environmental SEM (ESEM), this work visually examined the fabric of XG-treated kaolinite in dry and humid states. Based on visual observations, the compaction behavior and *s*_*u*_ of XG-treated kaolinites were analyzed to analyze the impact of XG on kaolinites’ fundamental properties.

### XRD at basal and prism peaks

**Figure 1:**
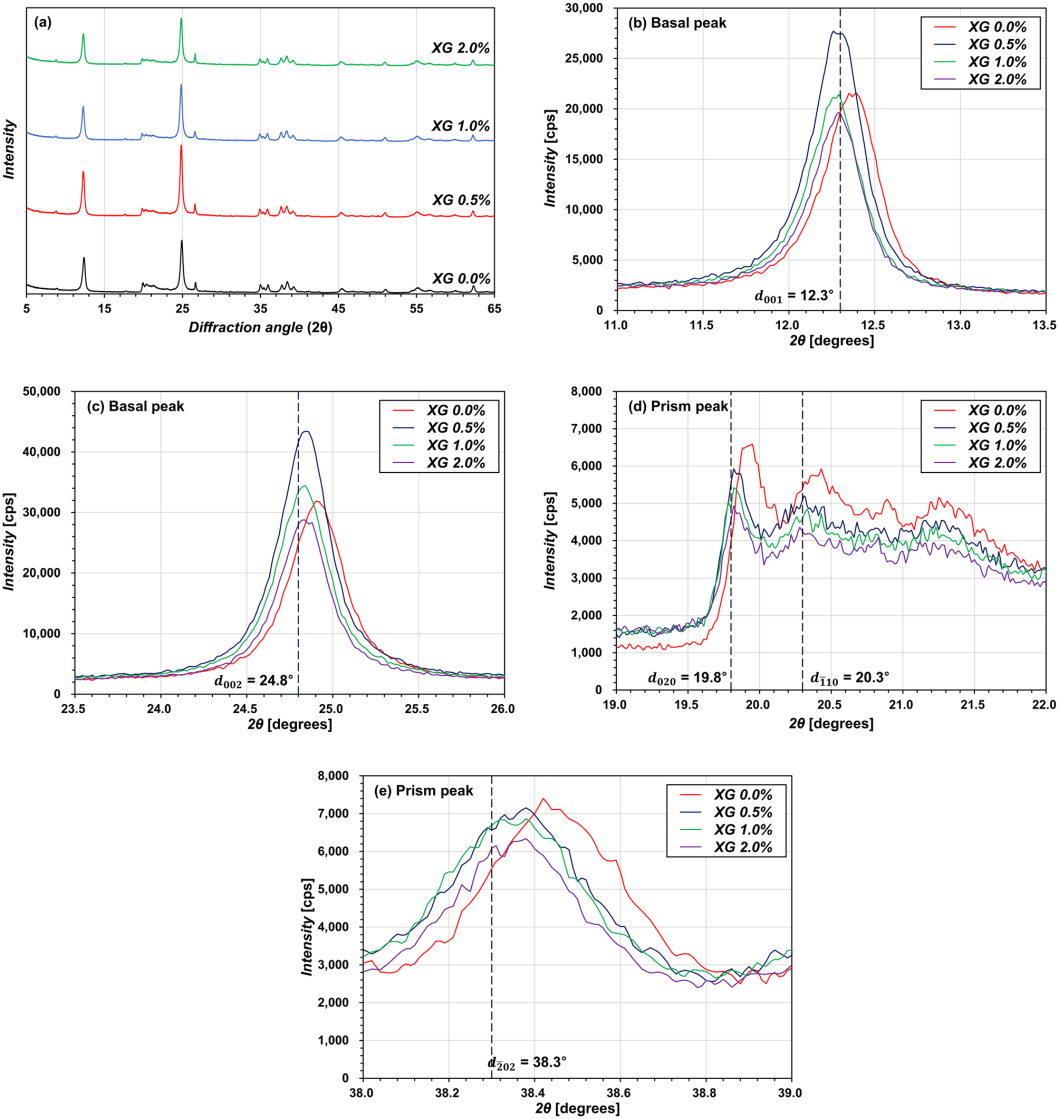
X-ray diffraction patterns of XG-treated kaolinite showing (**a**) at all diffraction angles, (**b**,**c**) at basal peaks, (**d**,**e**) at prism peaks.

Figure 1 depicts the XRD patterns of XG-treated kaolinites. The basic properties can be found in Extended Data Fig. 1 and Supplementary Table 1. The characteristic peaks are evident at diffraction angles in the order of 2θ = 12.31°, 24.84°, 20.34°, corresponding to the planes 001, 002, and 110 (Supplementary Table 2, and Extended Data Fig. 2). Here, the faces of the clay particles mostly consist of basal planes, and edges of clay particles mostly consist of prism planes^41^. The observed dominant basal peaks indicate that most clay particles are laminarly oriented, which is consistent with the report of Żbik et al.^42^. The surface charge of kaolinite primarily consists of silica facets, gibbsite facets, and edges. According to the reported isoelectric points of each surface^43–47^, the gibbsite facets and edges exhibit a positive charge, while the silica facets exhibit a negative charge within the pH range (5.3 ± 0.1) used in this study. Consequently, the negatively charged silica faces attract the positively charged gibbsite faces and edges, leading to the formation of face-to-face (FF) and edge-to-face (EF) fabric arrangements, respectively. The observed dominant basal peaks can be attributed to the higher surface area of the gibbsite faces compared to the edge surfaces.

**Figure 2:**
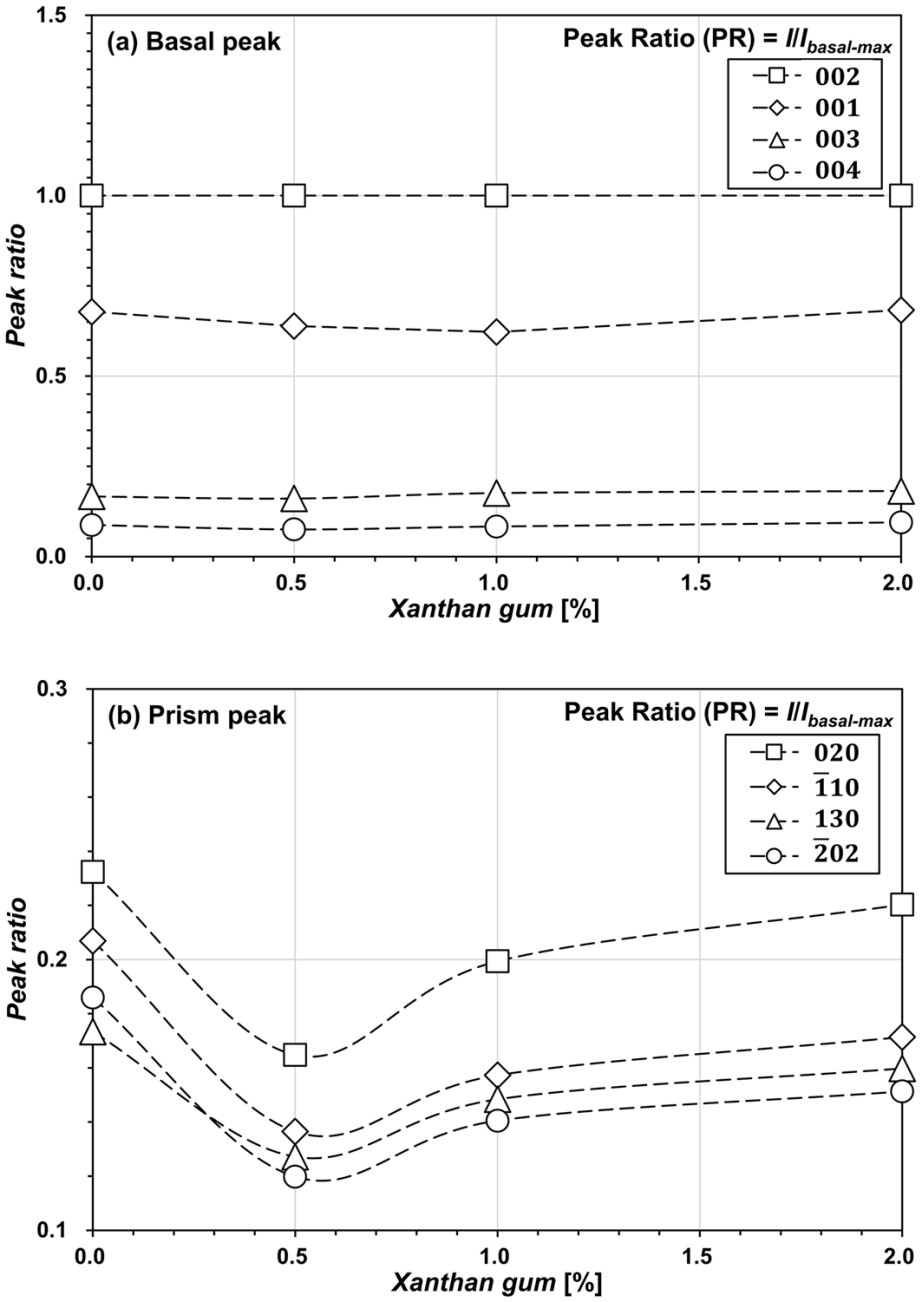
Variation in peak ratio with XG content at (**a**) basal and (**b**) prism peaks.

There is no major shift in the peak angle due to XG, indicating that the addition of XG in kaolinite has no substantial impact on the interlayer spaces between silicate layers as intercalation and exfoliation^48^. Instead, the XG content used in this study serves as a conventional composite filler, thus affecting the tactoids comprising several stacked silicate monolayers^49^. The XG-treatments change the intensity of basal and prism peaks. Kaolinites with 0.5% and 1.0% of XG-treatments showed a higher intensity of the basal peaks than the untreated kaolinite (Fig. 1b,c); however, all XG-treated kaolinites exhibited a reduction in the strength of the prism peaks compared to that of the untreated kaolinite (Fig. 1d,e). The change in peak strengths indicates that the XG affected the kaolinite properties because it transformed their fabrics^50,51^. Previous studies have highlighted the role of XG in promoting fabric transformation in kaolinite through its unique interaction with the clay surface, resulting in significant alterations in the hydro-mechanical properties of the clay^11,52^.

### Effect of XG on peak ratio

For a quantitative analysis of the variations in kaolinite fabrics with XG content, the relative intensities of each peak and the highest basal peaks—that is, the peak ratio (*PR*)—can be computed as follows^53^:

1 $$PR{ } = { }I_{{2\uptheta }} /I_{{{\text{max}}}} ,$$

where *I*_2*θ*_ denotes the intensity recorded at a diffraction angle of 2*θ*, and *I*_*max*_ denotes the specimen’s strongest intensity—that is, the 002 planes in this study. The results showed that the addition of XG had a negligible impact on the basal peaks (Fig. 2a) but reduced the peak ratio at prism peaks (Fig. 2b). Owing to the positioning of prism planes at the edges of clay particles^54^, higher prism peaks indicate that the geometric arrangement of kaolinite clay platelets was randomly oriented, implying the formation of EF contacts. In contrast, the observed reduction in the peak ratio at prism peaks indicates a more FF particle arrangement^41^, due to the influence of XG. Furthermore, XG of *m*_*b*_/*m*_*s*_ = 0.5% exhibited the lowest PR at prism peaks, whereas the PR at prism peaks steadily increased with XG for *m*_*b*_/*m*_*s*_ > 0.5%.

### Effect of XG–clay fabrics on compaction behavior

**Figure 3:**
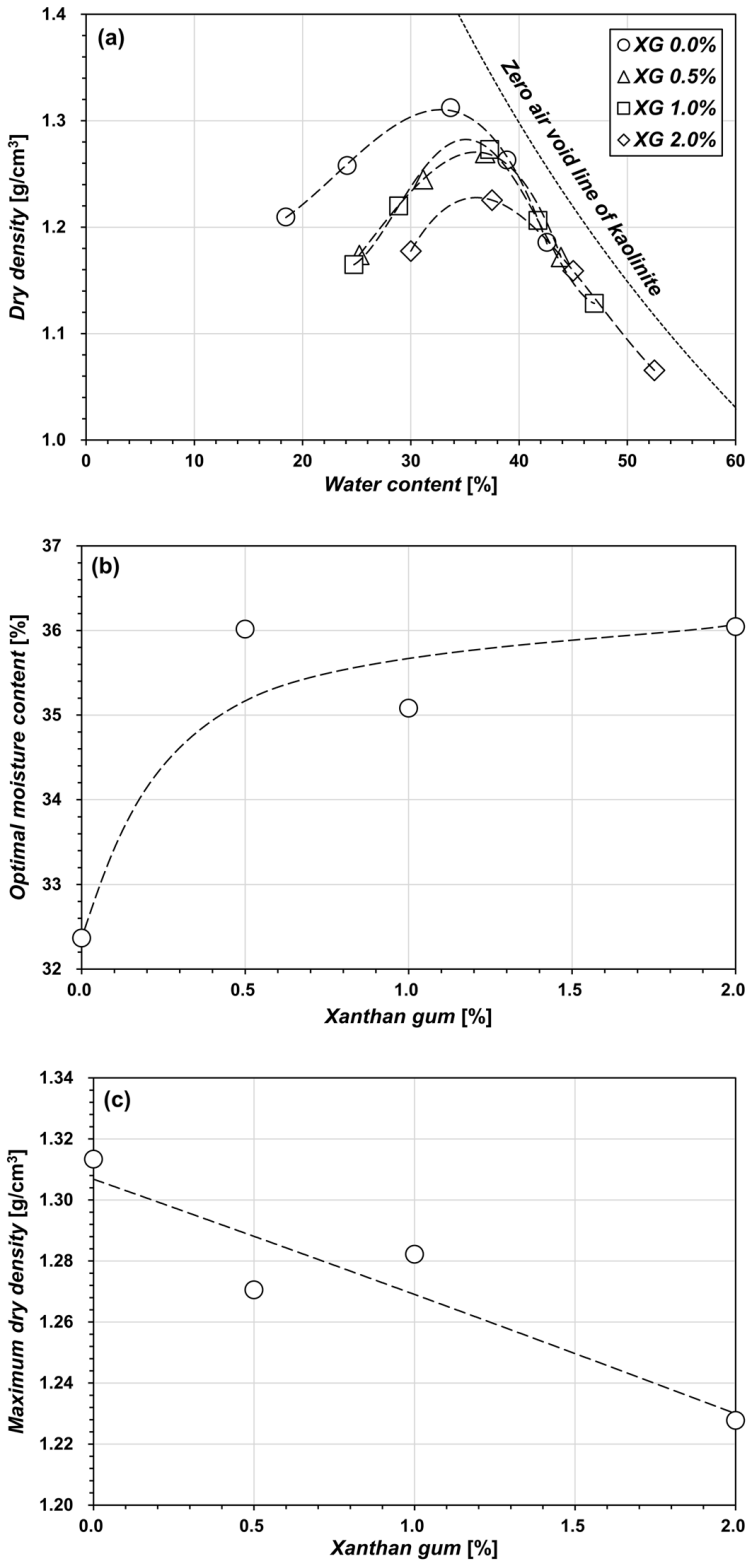
Compaction behavior of XG-treated kaolinite showing (**a**) the compaction curve, (**b**) the variation in optimal moisture content with XG, and (**c**) the variation in maximum dry density with XG.

When considering the building of pavements, airports, levees, and dams, compaction characteristics are essential parameters that affect the mechanical properties of soils^55^. Figure 3 shows the results of standard Proctor compaction tests conducted on XG-treated kaolinites under varying *m*_*b*_/*m*_*s*_ conditions. The compaction curve (Fig. 3a) shows that the XG decreases the variation in dry unit weight by *w*, which further indicates that XG reduces the soil’s sensitivity to variations in *w*^56^.

The XG raises the optimal moisture content (OMC) (Fig. 3b) and lowers the maximum dry density (MDD) (Fig. 3c). This tendency can be attributed to the formation of an interconnecting network—that is, hydrogen bonding or direct electrical bonding—between the kaolinite particles^10,57^, resulting in a modification of the kaolinite fabrics as indicated by the XRD results, which resist the compaction energy^16,56^. Ni et al.^58^ also investigated the increase in OMC and the decrease in MDD with XG, indicating that this alteration is caused by the clay content and biopolymer characteristics. In the case wherein kaolinite is used, when the XG content (*m*_*b*_/*m*_*s*_) is > 0.5%, the observed OMC slightly decreases and the MDD increases. This trend is consistent with the work of Kang et al.^59^, which demonstrated a backbone trend at *m*_*b*_/*m*_*s*_ = 0.1%. Specifically, *m*_*b*_/*m*_*s*_ of 0.5% increases the OMC from 32.4% (untreated kaolinite) to 36%; however, the extra XG has a negligible impact on the OMC. Additionally, the MDD decreases as XG changes from 1.31 g/cm^3^ (untreated kaolinite) to 1.22 g/cm^3^ (*m*_*b*_/*m*_*s*_ = 2.0%). These results match the previous findings that XG with *m*_*b*_/*m*_*s*_ = 0.5% exhibit the local maximum liquid limits^11,52^ and *s*_*u*_^10^.

### Effect of XG on *s*_*u*_ of kaolinite at MDDs

**Figure 4:**
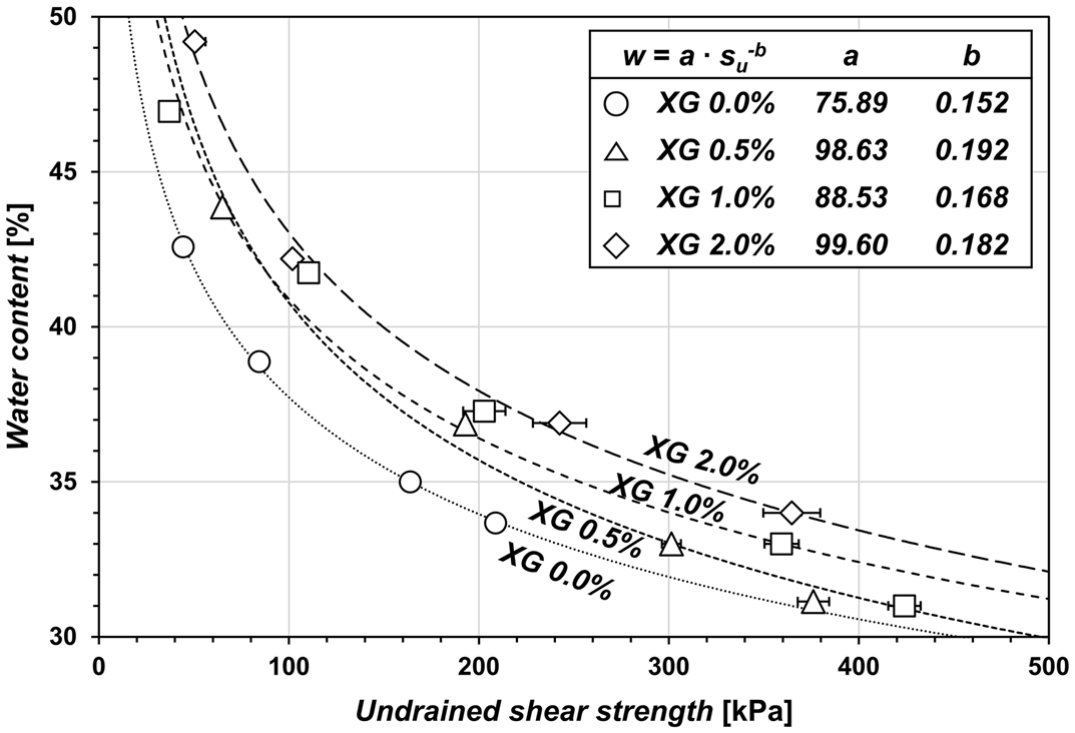
Effect of XG on the undrained shear strength of compacted kaolinite.

Figure 4 shows the vane shear test results of XG-treated kaolinites in terms of *s*_*u*_. At comparable water content, XG-treated kaolinites exhibit a higher *s*_*u*_ compared to untreated kaolinites. Based on the exponential relationship between *w* and *s*_*u*_^60^, the results of the vane shear tests can be reduced to the parameters *a* and *b*, where parameter *a* denotes *w* at *s*_*u*_ = 1 kPa, while parameter *b* denotes the variation of *w* in response to a change in *s*_*u*_. Parameters *a* and *b* increase with XG of *m*_*b*_/*m*_*s*_ = 0.5%, suggesting that XG enhances the *s*_*u*_ at the same *w* while reducing its sensitivity to *w* fluctuations. Then, parameters decreased as XG content varied from 0.5 to 1.0%, and they increased again with XG treatment *m*_*b*_/*m*_*s*_ > 1.0%.

### Microstructures and particle associations: based on microscopy observations

The visual inspection of XG-treated clays shows the interaction between kaolinite and XG, which affects the fabrics and mechanical properties of kaolinites. Notably, the fabric distinctions resulting from XG treatment were analyzed using the higher resolution capabilities of SEM. In contrast, ESEM was employed to investigate the behavior of kaolinite treated with XG under different relative humidity conditions, despite the challenges associated with image quality, specifically contrast and brightness, which are influenced by chamber humidity^61^. By employing both SEM and ESEM methods and considering their combined results, previous research has achieved consistent findings^62^.

**Figure 5:**
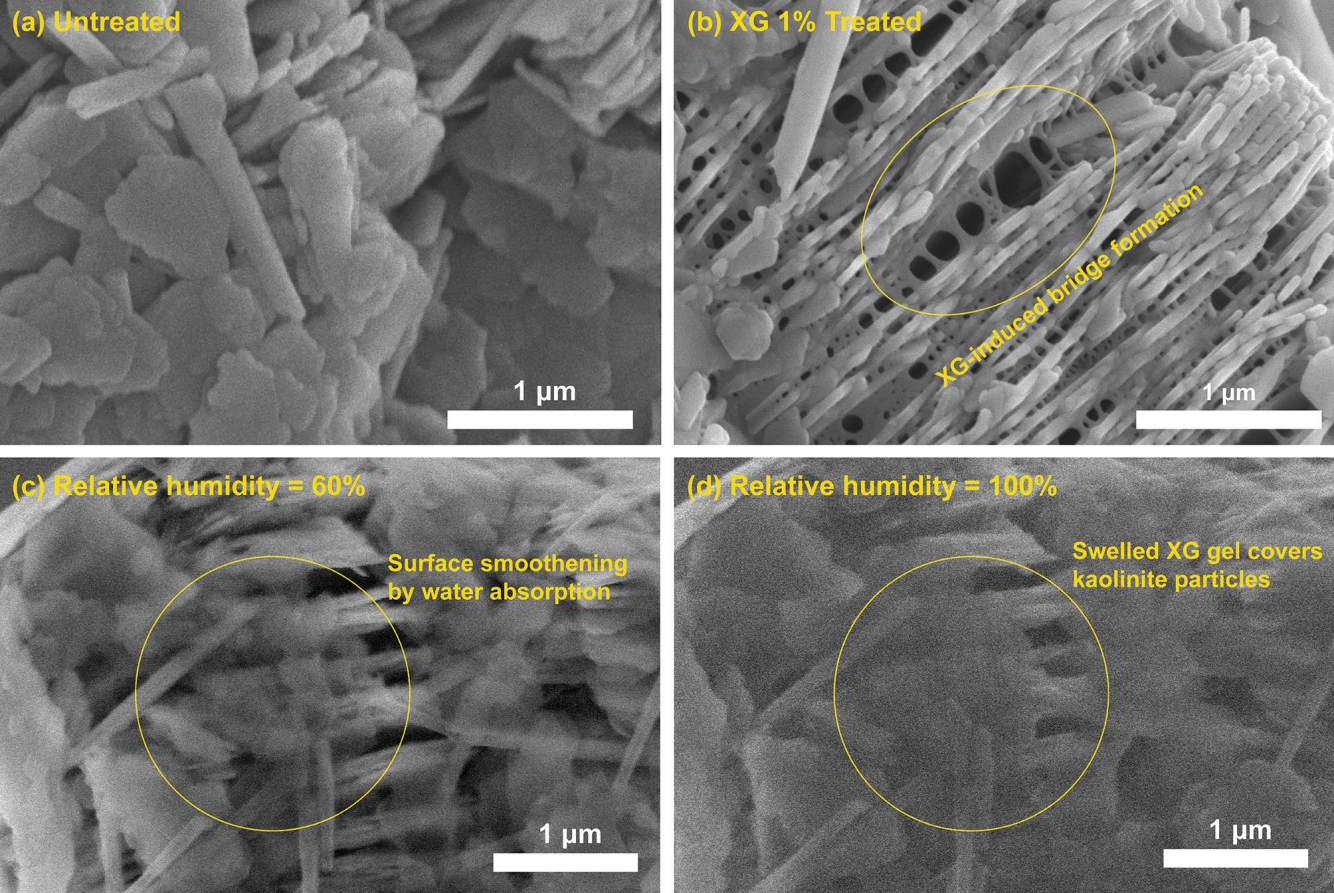
Microscopy observations of XG-treated kaolinites; SEM observations in a dry state for (**a**) untreated kaolinite and (**b**) XG 1%-treated kaolinite, and ESEM observations of XG 1%-treated kaolinite with a relative humidity of (**c**) 60%, and (**d**) 100%.

Compared to untreated kaolinite with random EE and EF associations (Fig. 5a), the XG in kaolinite produces interparticle bridges between kaolinite particles, inducing a higher degree of FF associations (Fig. 5b). Consequently, XG side chains interact with kaolinite via direct electrostatic attraction, ligand exchange, and hydrogen bonding^27,63^. Thus, the electrical interaction between XG and kaolinite particles leads to the creation of a bridge between particles^11^. When the XG powder is dissolved in water, the glucuronic and pyruvic acid groups on its side chains become active, and the molecule becomes negatively charged^52,64,65^. Under humid conditions, the XG side chain absorbs pore fluids and swells, resulting in its expansion. According to Dogan et al.^66^, XG absorbs approximately 25 g water per gram of XG. Consequently, XG forms bridges between the kaolinite particles, as shown in Fig. 5b. Additionally, XG absorbs the surrounding water molecules, as shown in Fig. 5c,d, leading to the generation of a viscous hydrogel around the kaolinite particles.

### Effects of XG on kaolinite fabrics

**Figure 6:**
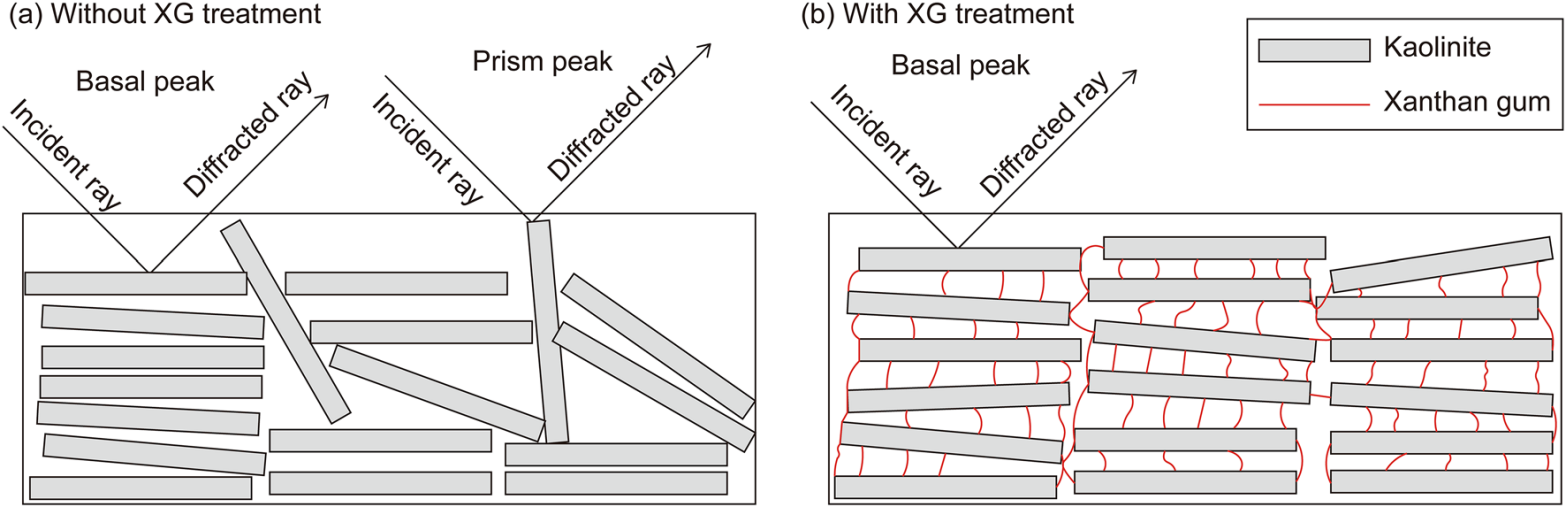
Kaolinite (**a**) without XG and (**b**) with XG.

It was found that XG affected the kaolinite particle associations. Based on the XRD and microscopy observations, Fig. 6 schematically shows the potential impact of XG on the kaolinite fabrics. In the absence of XG (Fig. 6a), kaolinite tends to flocculate in FF and EF contacts because of the net-attraction energy between the negatively charged face surfaces and the positively charged gibbsite and edge surfaces^63,67–69^. This formation of EF contacts leads to greater prism peaks than those of XG-treated kaolinites (Fig. 1).

In contrast, the XG-treated kaolinite form bridges between the kaolinite particles (Fig. 6b), which is evident in the microscopy images (Fig. 5b–d), resulting in more FF associations because of the electrostatic interaction with the kaolinite surface charges^11,52^. Consequently, the creation of XG-bridges peak at *m*_*b*_/*m*_*s*_ = 0.5%, demonstrating the minimal *PR* at prism peaks (Fig. 2b). Meanwhile, when *m*_*b*_/*m*_*s*_ is greater than 0.5%, the water absorption of the excess XG hydrogel hinders bridge formation^40^, leading to a higher *PR* at prism peaks. Notably, the *PR* at prism peaks for *m*_*b*_/*m*_*s*_ = 1.0–2.0% is still lower than that of untreated kaolinite.

### Effect of XG on mechanical properties of soil

In summary, the formation of XG-bridges and viscous XG hydrogels alters the mechanical properties of kaolinite soils. The XG-bridges between kaolinite particles changes the kaolinite fabrics into FF associations, and this further improves their resistance to compaction energy and shear stress. At the same time, the water absorption by XG also contributes to the mechanical properties of kaolinite clays. The excess XG in the void space primarily absorbs pore fluids and swells, which is evident in the ESEM observations (Fig. 5)—that is, swollen XG absorbs water, increasing the hydrodynamic volume and subsequently leading to a rise in the viscosity of the pore fluids^70^. Increasing pore-fluid viscosity is associated with cohesion, shear strength, damping ratio, and shear modulus^39,71,72^. Thus, factors such as XG-bridges, cause of fabric modification, and XG hydrogel fill the spaces between clay particles, leading to a reduction in the MDD (Fig. 3c), and an increase in OMC (Fig. 3b) and *s*_*u*_ (Fig. 4). Additionally, XG water absorption makes the kaolinite less susceptible to variations in *w*. Accordingly, XG reduces the fluctuations in dry density (Fig. 3a) and *s*_*u*_ with increasing *w* (Parameter *b* in Fig. 4).

Notably, XG content of *m*_*b*_/*m*_*s*_ = 0.5% exhibits a local minimum for XRD prism peaks (Fig. 2b) and a similar OMC with higher *m*_*b*_/*m*_*s*_ (e.g., 2.0%) (Fig. 3b), as well as an optimal biopolymer content for *LL*^11,52^. Regarding the aforementioned factors, it can be hypothesized that XG content (*m*_*b*_/*m*_*s*_) below 0.5% interacts primarily with the kaolinite surfaces, changing their fabrics. Despite the hindered XG fabric rearrangement observed in Figs. 1 and 5 for XG content exceeding 0.5%, *s*_*u*_ continues to increase with higher *m*_*b*_/*m*_*s*_. This indicates the significant role of XG's water absorption characteristics in enhancing the shear resistance of kaolinite. Rather than directly interacting with the kaolinite particles, XG content exceeding 0.5% primarily interacts with water molecules. Consequently, the plasticity index decreases as *m*_*b*_/*m*_*s*_ increases^11^. Notably, the plasticity index exhibits an inverse relationship with *s*_*u*_^60,73^. Furthermore, this behavior depends on the biopolymer and clay characteristics^23^. For instance, Chang and Cho^74^ reported that gellan gum biopolymer showed the maximum shear strength with 4% of biopolymer-to-kaolinite content. Even for the XG treatment, their effect differed by clay types^11^.

### Implications of XG treatment on the clay fabrics and resulting geotechnical properties

This study analyzed the impact of XG on the fabrics of kaolinite clays based on XRD analyses, compaction tests, laboratory vane shear experiments, and microscopy observations. The experimental results showed that XG modified the kaolinite fabrics through electrical charge interactions with the kaolinite surfaces. The XRD analyses showed that XG resulted in a decrease in the number of peaks at prism planes, indicating that XG altered the association of kaolinites from EF to FF. Moreover, the XRD patterns exhibited local peaks at *m*_*b*_/*m*_*s*_ = 0.5%. The SEM images also visually demonstrated XG-induced FF fabrics. In addition to XG-kaolinite interactions, the ESEM images indicated that the XG absorbed pore fluids and swelled. Evidently, the interactions between the XG, kaolinite, and water impacted the mechanical properties of the kaolinite clays. The XG-induced bridge formation between kaolinite particles enhanced the resistance of kaolinite to external forces. In void spaces, the XG absorbed pore fluids to produce viscous hydrogels. Owing to the complex interaction among XG, kaolinite, and pore fluids, the MDD decreased, and the OMC and *s*_*u*_ increased due to the XG treatment. A series of experimental analyses revealed that XG content (*m*_*b*_/*m*_*s*_) of 0.5% mainly affects the mechanical properties of kaolinite because it forms networks between kaolinite particles, while XG content (*m*_*b*_/*m*_*s*_) over 0.5% mainly absorbs pore fluids and increases the pore-fluid viscosity.

By investigating XG treatment effects on kaolinite, this study explores the potential applicability and performance of XG in geotechnical engineering applications. However, the unique mineral composition of kaolinite, characterized by its 1:1 layered tetrahedral and octahedral sheets, distinguishes it from other clay minerals with a 2:1 layered structure, such as illite and montmorillonite^75^. Owing to these distinct mineralogical characteristics, the specific properties associated with kaolinite may not directly apply to all engineering scenarios. Considering the diverse mineral composition of clays, future research should encompass the evaluation of other clay types to further enhance our understanding of their behavior and explore potential applications.

## Methods

### Materials: kaolinite, XG, and XG-treated kaolinite preparation

This study integrated kaolinite clay (Bintang, Indonesia) and XG biopolymer (CAS: 11138-66-2; Sigma Aldrich). Kaolinite—which comprises 1:1 layered tetrahedral and octahedral sheets—was chosen as a typical clay mineral because it is more responsive to fabric changes than 2:1 layered clay minerals^76,77^. Given kaolinite’s high width: thickness aspect ratio (3–18)^78–81^, the edge and face charge attraction is crucial for kaolinite fabrics and mechanical properties^82^. Moreover, kaolinite exhibits low cation adsorption because of the insignificant isomorphic substitution of Al^3+^ or Fe^3+^ for Si^4+^^78,83^. Although kaolinite possesses both pH-independent negative charges and pH-dependent charges, the overall charge characteristic becomes more positive at low pH values and more negative at high pH values^84^.

The XG is a biopolymer that is produced by the microbe *Xanthomonas campestris*, and its structure consists of repeated glucose units and side chains containing three sugar units^85^. The XG is a water-soluble biopolymer whose structure swells upon hydration via hydrogel bonding, the glucuronic (C_6_H_10_O_7_), and pyruvic acid (C_3_H_4_O_3_) groups on its side chain becoming negatively charged^52,64,65^. The XG is widely used as a thickening agent and a stabilizer for suspensions, solid particles, and foams^86,87^.

Supplementary Table 1 shows the index properties of kaolinite, indicating an increase in the *LL* and plastic limit (*PL*) with the addition of XG, as previously reported by Nugent, et al.^52^ and Chang et al.^11^. Bintang kaolinite is classified as clay with high plasticity (*CH*) according to the ASTM D2487 standard^88^. The kaolinite was dried in an oven at 110 °C to evaporate the pore fluids in clay matrixes prior to experiments, based on the ASTM D2216 standard^89^. In contrast, XG was used without pre-treatment. Extended Data Fig. 1 shows the particle size distribution curve of kaolinite and XG obtained using laser diffraction spectroscopy (Model HELOS/KR-H248, Sympatec GmbH, Clausthal-Zellerfeld, Germany) based on the ASTM D4464-15 and ASTM B822-20 standards^90,91^. The kaolinite particle shape was observed by scanning electron microscopy (Model SU5000, Hitachi, Tokyo, Japan).

The oven-dried kaolinite was mixed with XG powder at XG to the dry soil ratio in mass (*m*_*b*_/*m*_*s*_) of 0% (untreated), 0.5%, 1.0%, and 2.0%. Then, deionized water (DI) with pH of 6.2 ± 0.2 at 20 °C, with its designated water content (*w*), was added and thoroughly mixed until forming a uniform XG–clay–DI mixture. The pH of kaolinite was determined to be 5.3 ± 0.1 at 20 °C, following the guidelines outlined in the ASTM standard^92^.

### XRD analysis

The XG-treated kaolinites (*m*_*b*_/*m*_*s*_ = 0%, 0.5%, 1.0%, and 2.0%) with a *w* of 35% (around the optimal moisture content (OMC)) were adhered to 18 mm × 18 mm cover glass (Marienfeld; Germany) and air-dried for 24 h at 20 °C. The XRD patterns of the XG-treated kaolinites were then collected using Cu K-alpha radiation of wavelength 0.154 nm, which were generated using a Rigaku SmartLab diffractometer (Rigaku, Japan) operated by a 9 kW X-ray generator.

Because the diffraction angle is a mineral-dependent feature, XRD techniques have been used to identify minerals^93^. The XRD measures the number of diffracted X-rays that impact a crystal of clay particles and outputs the clay textile identification based on Bragg's law—that is, *nλ* = 2*d*·sin*θ*; here, *n* denotes the order of reflection, *λ* denotes the wavelength, *d* denotes the particle spacing, and *θ* denotes the diffraction angle.

This study analyzed the XG-treated kaolinite fabrics in accordance with the methodologies proposed by Sachan and Penumadu^41^, wherein pure-kaolinite fabrics were recognized based on the basal and prism peaks of the XRD patterns as shown in Supplementary Table 2. Extended Data Fig. 2 depicts the basal and prism planes of a unit cell of kaolinite. The parallel orientation of platelets—that is, the FF associations—favors basal planes on the surface, resulting in a higher count at basal peaks. In contrast, the honeycomb orientation of platelets—that is, the EF associations—increases prism reflections and decreases basal planes.

### Compaction tests

The compaction characteristics of XG-treated kaolinite clay (*m*_*b*_/*m*_*s*_ = 0%, 0.5%, 1.0%, and 2.0%) with a *w* of 15%–55% were analyzed using a standard Proctor compaction test. The XG-treated kaolinite mixtures were placed in a 0.95-l cylindrical mold and compacted by following the ASTM D698-12E2 standard. After compaction, the surface was flattened using a spatula. The final total soil weight was obtained. Next, the laboratory vane shear apparatus measured the *s*_*u*_ of compacted kaolinites.

### Laboratory vane shear tests

After compaction, laboratory vane shear tests were conducted to analyze the XG-affected kaolinite fabrics (*m*_*b*_/*m*_*s*_ = 0%, 0.5%, 1.0%, and 2.0%) on the *s*_*u*_ of kaolinite. The *s*_*u*_ of XG-treated kaolinites was determined by rotating a rectangular vane (width = 12.7 mm, height = 12.7 mm, and thickness = 0.05 mm) at the top, center, and bottom of the compacted specimen at a velocity of 60˚/min based on the ASTM D4648-05 standard^95^. Additionally, experiments were conducted in triplicate for each specimen to increase the reproducibility of the experimental data. Following the vane shear tests, the average *w* at each tested position was measured according to the ASTM D2216 standard^89^.

### Microscopic analysis: SEM and ESEM

This study observed the microscale interactions between XG and kaolinite under dry and humid conditions, the relative humidity being 60% and 100%, respectively, using SEM (SU-5000, Hitachi High Technologies) and ESEM (Model Quattro ESEM, Thermo Fisher Scientific Inc., Waltham, USA). Untreated and treated kaolinites—that is, *m*_*b*_/*m*_*s*_ = 0, 1.0%—at *w* = 35% were air-dried for 24 h at 20 °C and attached to a 25-mm diameter SEM mount using carbon conductive tabs (PELCO Tabs; Ted Pella, Inc.). Before SEM observations, the specimens were osmium (OsO_4_)-coated for 10 s under a vacuum using a plasma coater (OPC-60A).

The ESEM, which controls the water vapor pressure (10–4000 Pa) and the relative humidity in its specimen chamber^96^, was used to assess the variation of XG-treated kaolinite (*m*_*b*_/*m*_*s*_ = 1%) using a change in the relative humidity. The samples (*w* = 35%) were initially attached to an ESEM mount, and the specimen surfaces were exposed to the electron beams thereafter. During the observations, the relative humidity fluctuated between 0 and 100%.

## Supplementary Information

Supplementary Information.

## Data Availability

The data that support the findings of this study are available from the authors upon reasonable request.

## Abbreviations

BPST: Biopolymer-based soil treatment
*LL*: Liquid limit (%)
*PL*: Plastic limit (%)
*s*_*u*_: Undrained shear strength (kPa)
EF: Edge-to-face association
FF: Face-to-face association
SEM: Scanning electron microscopy
TEM: Transmission electron microscopy
XG: Xanthan gum
ESEM: Environmental scanning electron microscopy
*D*_50_: Mean particle diameter (μm)
*SSA*: Specific surface area (m^2^/g)
*CEC*: Cations exchange capacity (meq/100 g)
*m*_*b*_/*m*_*s*_: XG-to-dry soil ratio in mass (%)
*w*: Water content (%)
OMC: Optimal moisture content (%)
MDD: Maximum dry density (g/cm^3^)
DI: Deionized water
XRD: X-ray diffraction analysis
PR: Relative intensities of each XRD peak and the highest basal peaks (–)
*a*: Water content at the undrained shear strength of 1 kPa (%)
*b*: Variation of water content at a change in the undrained shear strength (–)
