# Supplementary material for: Xanthan biopolymer-based soil treatment effect on kaolinite clay fabric and structure using XRD analysis
Source: Sci Rep. 2023 Jul 19;13:11666. doi: 10.1038/s41598-023-38844-w (PMC10356783; doi:10.1038/s41598-023-38844-w)
Supplement: Supplementary file 1 — Supplementary Information. [file 41598_2023_38844_MOESM1_ESM.docx]

**List of Extended Data Figs**

**Extended Data Fig. 1.** Material properties used: (a) particle size distribution curve, and (b) particle morphology of kaolinite.

**Extended Data Fig. 2.** Planes in a unit cell of kaolinite: (a) basal planes and (b) prism planes.

| 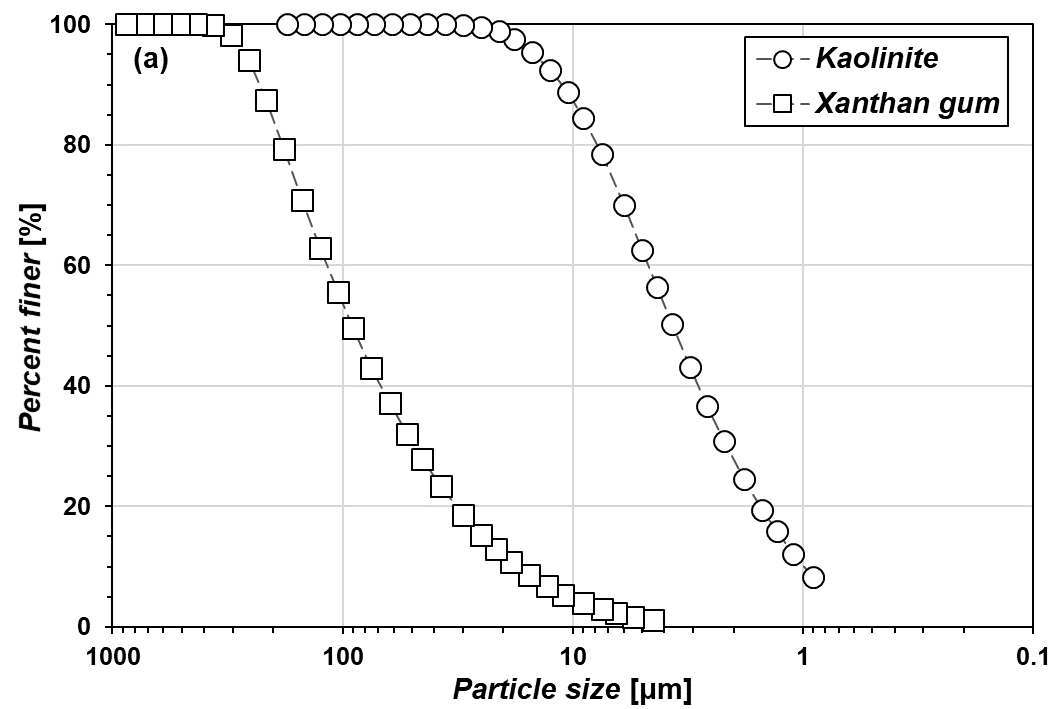 |
| --- |
| 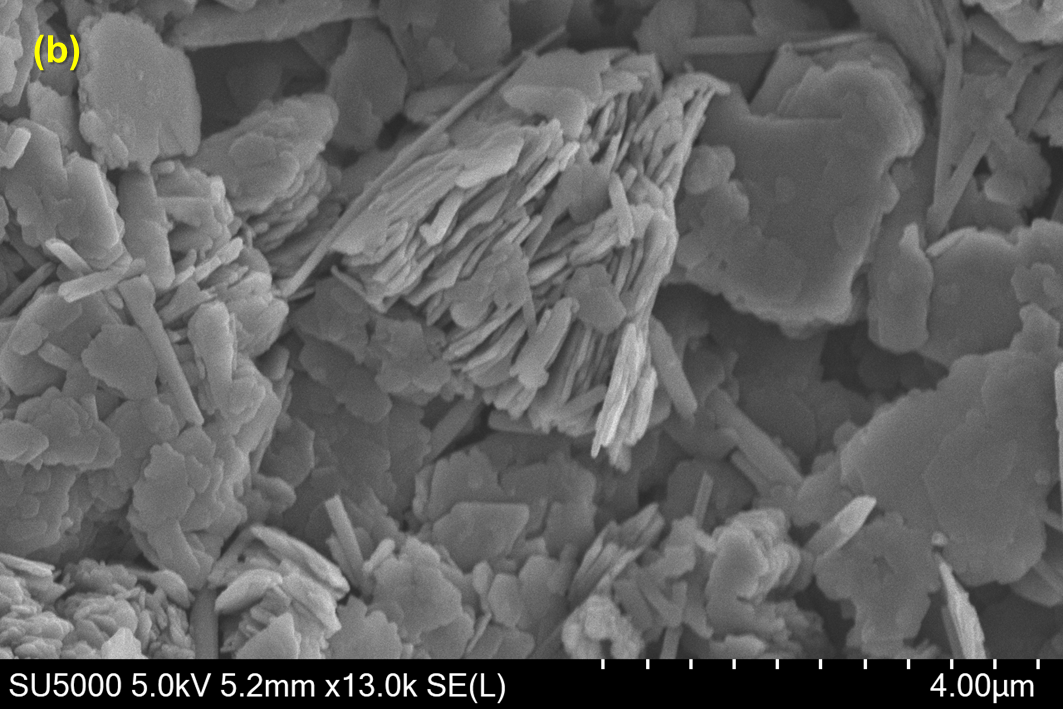 |
| **Extended Data Fig. 1.** Material properties used: (a) particle size distribution curve, and (b) particle morphology of kaolinite. |

| 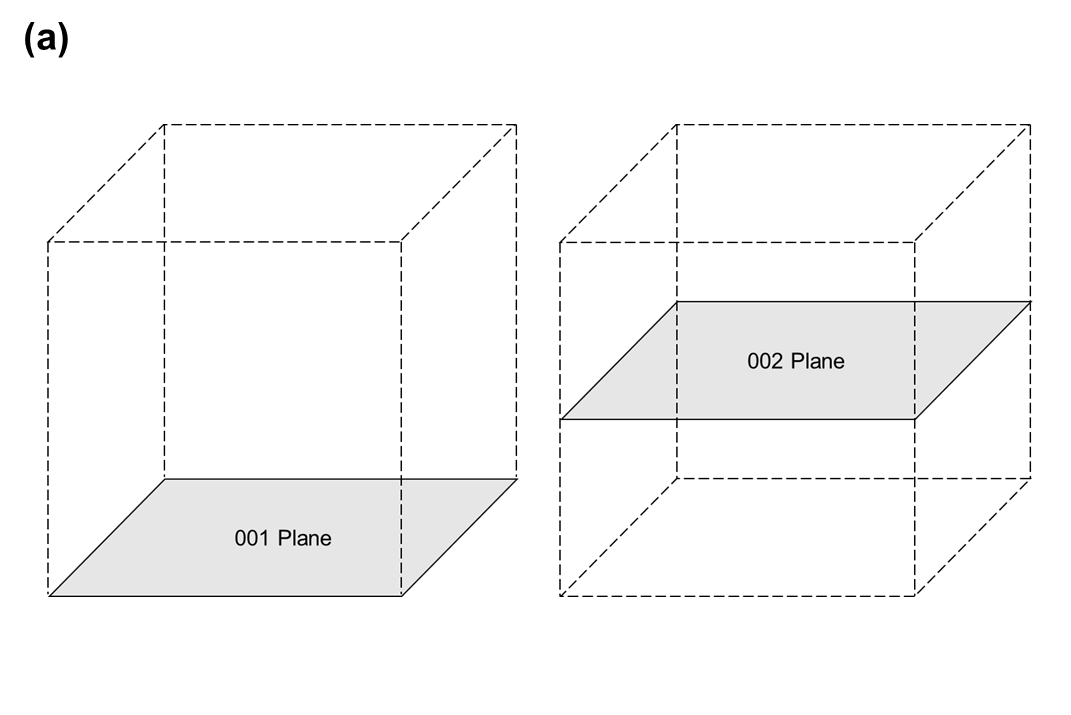 |
| --- |
| 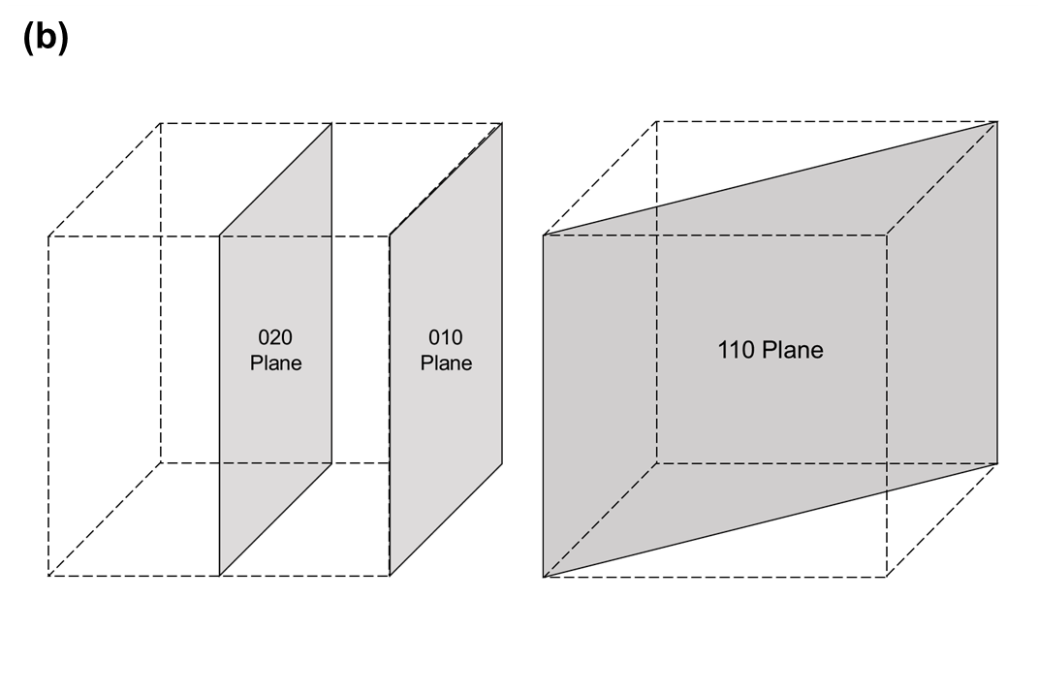 |
| **Extended Data Fig. 2**. Planes in a unit cell of kaolinite: (a) basal planes and (b) prism planes. |

**List of Supplementary Tables**

**Supplementary Table 1**. Index properties of the kaolinite used in this study.

**Supplementary Table 2**. Basal and prism peaks in the XRD patterns of kaolinite (Sachan and Penumadu ^1^).

**Supplementary Table 1**. Index properties of the kaolinite used in this study.

| ***D_50_***  **[μm]** | ***SSA***  **[m^2^/g]^1)^** | ***CEC***  **[meq/100 g]^2)^** | **pH^3)^** | **XG**  **[%]** | **Deionized water** | |
| --- | --- | --- | --- | --- | --- | --- |
|  |  |  |  |  | ***PL***  **[%]** | ***LL***  **[%]** |
| 3 | 22 | 6.1 | 4.2 | 0 | 31 | 70 |
|  |  |  |  | 0.1 | 35 | 74 |
|  |  |  |  | 0.5 | 32 | 97 |
|  |  |  |  | 1 | 40 | 80 |
|  |  |  |  | 2 | 36 | 79 |

**Note**: ^1)^ Specific surface area obtained using methylene blue adsorption measurement ^2^, ^2)^ Cation exchange capacity measured using methylene blue cation exchange capacity ^3^, ^3)^ Supernatant pH measured after settling sediment with 300% water content.

**Supplementary Table 2**. Basal and prism peaks in the XRD patterns of kaolinite ^1^.

| **Type of peaks** | **Peaks** | **2θ (degrees)** |
| --- | --- | --- |
| Basal peaks | 001 | 12.3 |
|  | 002 | 24.8 |
|  | 003 | 37.6 |
|  | 004 | 51.1 |
| Prism peaks | 020 | 19.8 |
|  | 110 | 20.3 |
|  | 130 | 35.1 |
|  | 202 | 38.3 |

**Related References**

1 Sachan, A. & Penumadu, D. Identification of microfabric of kaolinite clay mineral using X-ray diffraction technique. *Geotechnical and Geological Engineering* **25**, 603-616, doi:<http://dx.doi.org/10.1007/s10706-007-9133-8> (2007).

2 Santamarina, J. C., Klein, K. A., Wang, Y. H. & Prencke, E. Specific surface: determination and relevance. *Can. Geotech. J.* **39**, 233-241, doi:<https://doi.org/10.1139/t01-077> (2002).

3 Inglethorpe, S. D. J., Morgan, D. J., Highley, D. E. & Bloodworth, A. J. Industrial minerals laboratory manual: Bentonite. 125 (British Geological Survey, Nottingham, UK, 1993).
